# Dynamic-SQL: an adaptive NL2SQL framework with multi-path fusion reasoning and execution feedback correction

**DOI:** 10.1038/s41598-026-47693-2

**Published:** 2026-04-15

**Authors:** Hongbin Hao, Xin Zheng, Xuhong Yu

**Affiliations:** 1 Guizhou Key Laboratory of Advanced Computing, Guizhou Normal University, Guiyang, 550001 China; 2 School of Cyber Science and Technology, Guizhou Normal University, Guiyang, 550001 China

**Keywords:** NL2SQL, LLM, Schema linking, Hybrid vector retrieval, Multi-path fusion reasoning, Self-correction, Engineering, Mathematics and computing

## Abstract

Current NL2SQL systems degrade sharply when confronted with practical constraints such as limited prompt length and the inability to fine-tune large language models (LLMs). Performance drop is especially pronounced in complex databases, where inaccurate schema linking, vague value conditions, and weak self-correction dominate the error surface. We propose Dynamic-SQL, an adaptive framework that couples multi-path chain-of-thought fusion with execution-based feedback correction. A dense–sparse hybrid vector space is first constructed to dynamically retrieve relevant schema elements, and an LLM is leveraged to generate an explicit schema subgraph. Real-value and few-shot exemplars are then injected to enrich the prompt and sharpen value conditioning. Multiple candidate SQL statements are produced via diverse reasoning paths; their chains of thought are fused to cover latent semantic interpretations, and execution feedback is exploited for iterative self-correction until convergence. On the BIRD benchmark, Dynamic-SQL, powered by the open source qwen2.5-coder-32b-instruct, reduces the average prompt length by **50.83%**, raises strict schema-linking recall from **72.63%** to **90.66%**, and achieves **63.23%** execution accuracy. By systematically addressing schema linking, exemplar augmentation, multi-path fusion reasoning, and self-correction, the framework offers a transferable paradigm for deploying LLMs in complex database querying scenarios.

## Introduction

NL2SQL aims to automatically translate natural-language queries into executable SQL statements, enabling non-expert users to access structured data without learning SQL syntax^1^. In addition to accurately capturing the intent of a query, an NL2SQL system must fully understand the underlying database–its schema, inter-table relationships, and attribute semantics–to ensure both syntactic and semantic correctness of the generated SQL.

Recent progress in large language models (LLMs) has spurred a surge of prompt-engineering and fine-tuning methods that achieve impressive results on public NL2SQL benchmarks^2^. Yet, three practical hurdles remain. First, prompt-length limits prevent a complete database schema from being included, leading to inadequate semantic coverage or excessive noise^3^. Second, data-privacy or resource constraints often make it impossible to fine-tune the LLM in production^4^. Third, high-performance proprietary models (e.g., GPT-4) are costly, rate-limited, and subject to restrictive privacy policies, hindering large-scale deployment^5^. Consequently, building a high-performance NL2SQL framework that relies solely on open-source models and requires no fine-tuning has become a pressing research topic for practical LLM applications.

In existing work, the quality of the information injected into the prompt directly determines SQL accuracy. Supplying the database schema, the user query, and a few in-context examples enables an LLM to produce reasonably correct SQL for simple databases^6^. However, real-world information-management systems typically contain large, highly interconnected tables, and a single query typically touches only a small subset of them. Feeding the entire schema inflates computational costs and introduces substantial noise. Current research therefore relies on *schema linking* to identify and extract a semantic sub-schema relevant to the query^7^. Despite its value in reducing input redundancy, schema linking still faces two key challenges in practice. First, linguistic ambiguity in natural language makes column matching uncertain. Second, field names and values in business databases are often abbreviated or non-standard, further complicating semantic alignment^8^. For instance, a “gender” column may be stored as sex, gender, or gender_id, with values such as “0”, “m”, or “F”, whereas users are more likely to mention “female” or “woman”. Such inconsistencies dramatically degrade SQL validity and executability.

Ontology alignment has long been an essential technique in the domain of data integration, where the goal is to reconcile disparate data sources by aligning their schemas, fields, and semantic relationships. Early work in this area, such as by Melnik^9^, focused on generic model management approaches that could align database schemas and queries without relying on inference-based methods. These ontology-driven methods provide reliable schema matching but tend to be more rigid and less adaptive to complex or evolving databases.

In contrast, recent work has explored the integration of machine learning into ontology alignment. For instance, Hao et al.^10^ proposed embedding-based ontology alignment approaches, which use semantic and structural embeddings to enhance schema matching. These learning-based approaches have shown promising results in capturing more nuanced relationships between schemas and queries but are still limited by the availability and quality of training data. The learning-based models are also prone to errors when dealing with inconsistent or non-standard field names in real-world databases.

To address these challenges, we propose Dynamic-SQL, an adaptive framework designed to enhance schema linking under tight prompt budgets, mine information directly from database contents to disambiguate user queries, and boost SQL accuracy through multi-path chain-of-thought fusion and execution feedback. Specifically, we first build a *dense–sparse hybrid vector space*: dense vectors capture semantic similarity, while sparse vectors reinforce exact value matching^11^. Using items retrieved from this hybrid space, an LLM generates an initial SQL statement, which is then reverse-parsed to extract essential elements. Foreign-key metadata are used to complete join paths, forming a semantically coherent sub-schema. We then augment the context with real-value examples retrieved via the sparse space, explicitly constraining entity and condition interpretation, and embed highly relevant question–and–answer pairs as few-shot exemplars to guide the LLM toward the correct structural pattern and semantic boundary. Candidate SQLs produced under different schema contexts are combined through multi-path chain fusion. Finally, an iterative self-correction module refines the SQL based on execution feedback.

Early research on Text-to-SQL was largely based on carefully designed rules and templates^12^. These methods performed well in simple database scenarios but became increasingly difficult to design as database environments grew more complex and the cost of manual rule creation increased^13,14^. Due to the inherent similarity between the Text-to-SQL task and machine translation, many researchers have applied Seq2Seq models to handle the task^15^. These methods typically consist of two stages: the encoder, which understands the user’s natural language query and the database structure (e.g., table names, column names), and the decoder, which generates the corresponding SQL query. Examples include LSTM-based encoder-decoder structures^16^, Transformer architectures^17^, and generation strategies such as sketch generation or end-to-end generation.

With the success of pre-trained language models (PLMs) in natural language processing tasks, more and more research has incorporated PLMs into the Text-to-SQL task, significantly improving the model’s ability to handle complex queries and multi-table relationships^18^. These PLM-driven methods not only offer better expressiveness in modeling the semantic alignment between natural language and database schemas but also push performance further through mechanisms like table content encoding and pre-training. Recently, the rise of large language models (LLMs) has further accelerated progress in Text-to-SQL research. For example, methods like MAC-SQL^19^, C3^20^, and DIN-SQL^21^ employ chain-of-thought (CoT)^22^ multi-step reasoning frameworks, combining task decomposition and reasoning enhancement strategies with LLMs, leading to substantial improvements in handling complex queries. Other studies generate multiple candidate SQL queries and select the best one based on a selection strategy^23,24^. For instance, Chase-SQL^25^ guides LLMs to generate multiple SQL candidates through different prompts or diverse context settings, and selects the optimal query, effectively reducing the impact of single prediction errors.

Schema linking is a critical step in the Text-to-SQL task, aimed at identifying the mapping between database tables, columns, and the query semantics. Early methods mainly relied on simple string matching or rule-based engines, but their effectiveness was limited when dealing with semantically rich or variant expressions. To address this, methods like RAT-SQL^26^, SchemaGNN^27^, and ShadowGNN^28^ introduced relation-aware self-attention mechanisms to model the complex dependencies between natural language and database schemas using graph neural networks. SADGA^29^ further proposed a dual-graph encoding framework that models structural information from both natural language queries and database schemas and achieves semantic fusion through interaction. In recent years, as LLMs have shown strong capabilities in natural language understanding, more research has attempted to incorporate them into the schema linking task. For example, the CHESS^30^ method improves schema linking accuracy by retrieving relevant information from the database schema and specific values. RSL-SQL^31^ proposed a bidirectional linking approach to enhance schema matching ability.

We conduct extensive experiments on the BIRD^32^ benchmark. With the open-source models *qwen2.5-coder-32b-instruct* and *qwen2.5-coder-7b-instruct*, Dynamic-SQL achieves execution accuracies of 63.23 % and 50.65 %, respectively. Ablation studies confirm the positive contribution of each module. During schema linking, the hybrid retrieval plus LLM-generation strategy cuts average prompt length by 50.83 % while raising strict recall for *qwen2.5-coder-32b-instruct* from 72.63 % to 90.66 %, demonstrating clear superiority.

In summary, our main contributions are:

1. A hybrid schema-linking strategy that combines dense–sparse retrieval with LLM generation, reducing noise while preserving key elements and compressing prompt length.
2. A context-enhancement module that injects real-value examples and structurally similar Q&A pairs, and a multi-path fusion reasoning framework that strengthens value awareness and semantic mapping.
3. An execution-driven iterative self-correction process that improves the adaptability and practicality of open-source LLMs in complex databases.
4. Comprehensive experiments showing that Dynamic-SQL, when paired with *qwen2.5-coder-32b-instruct*, outperforms many GPT-4–based methods, exhibiting strong generalization, portability, and deployment potential.

## Results

### Overall framework

**Fig. 1:**
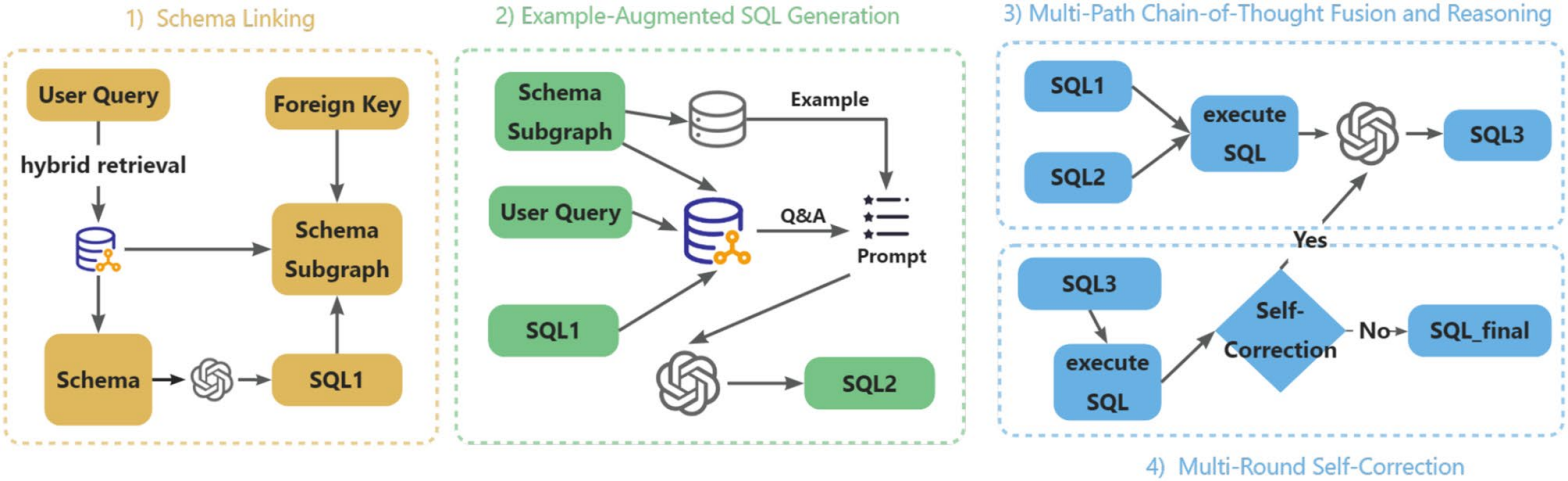
Dynamic-SQL Framework Overall Workflow Diagram.

This section outlines the overall process of the Dynamic-SQL framework, which is built around prompt compression, structural understanding, and execution feedback. As shown in Fig. 1, the framework consists of four key stages: Schema Linking, Example-Augmented SQL Generation, Multi-Path Chain-of-Thought Fusion and Reasoning, and Multi-Round Self-Correction. The goal of these stages is to improve the accuracy, interpretability, and adaptability of the generated SQL queries, as summarized in the step-by-step process of generating the final SQL query using this framework:

1. **Schema Linking (***SL***):** By leveraging dense and sparse vectors for hybrid retrieval, combined with the reasoning capabilities of large models, this stage accurately maps the semantic information in the natural language query to the tables and column fields in the database. It constructs a sub-schema highly relevant to the query’s intent, providing structured prior knowledge for subsequent SQL generation.
2. **Example-Augmented SQL Generation (***EASG***):** Based on the schema linking results, this stage introduces real-value examples related to the query intent, enhancing the contextual constraints in the prompt. It explicitly improves the LLM’s ability to perceive value conditions, while selecting high-quality question-answer pairs with structures and semantics highly similar to the current query as few-shot examples. These examples are embedded into the prompt, guiding the LLM to generate SQL queries with more comprehensive semantic coverage and a more reasonable structure.
3. **Multi-Path Chain-of-Thought Fusion and Reasoning (***MPFR***):** This stage takes the candidate SQL queries generated in the previous two stages as input. By combining database execution feedback with a chain-of-thought reasoning mechanism^33^, the method compares the structural and execution differences between the candidates. The fusion process produces a final SQL query with higher semantic consistency and execution accuracy.
4. **Multi-Round Self-Correction (***MRS***):** Based on the execution results of the SQL query generated in the third stage, the self-correction mechanism is triggered. If issues such as execution failure or empty results occur, an iterative optimization process is initiated. This process involves rollback reconstruction and multi-path chain fusion strategy, gradually converging towards a semantically aligned, syntactically correct, and highly executable SQL query.

Below is the pseudocode that describes the overall framework of Dynamic-SQL, providing a structured view of each stage in the process:

**Algorithm 1:**
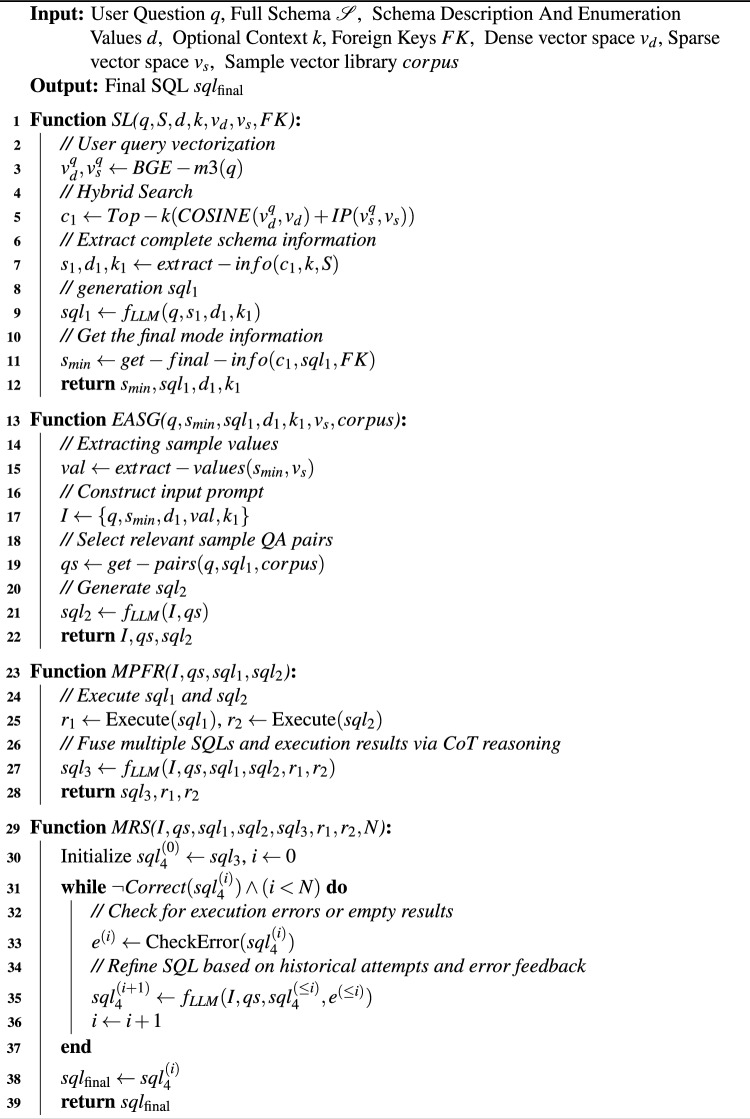
Dynamic-SQL Framework

### Dataset

The experiments in this paper are conducted on the open-source BIRD dataset. In the NL2SQL field, the BIRD dataset has demonstrated significant academic value due to its scale, complexity, and domain diversity, aligning well with the real-world challenges emphasized in this study. During the annotation process, BIRD adopts a double-blind protocol to ensure semantic consistency and logical correctness. It also provides complete database schemas and executable environments, which substantially enhance the realism and practical value of model evaluation. However, the performance of state-of-the-art methods on BIRD still falls short of human-level capabilities, particularly in terms of execution feasibility and semantic generalization, further highlighting the dataset’s difficulty and research potential.

### Evaluation metrics

To comprehensively evaluate the performance of Dynamic-SQL in complex database environments, this paper adopts the following three evaluation metrics:

Execution Accuracy (EX): This metric is defined as the proportion of samples for which the predicted SQL queries yield exactly the same execution results as the ground-truth SQL queries^34^.

Recall: To further assess the framework’s performance in the schema linking stage, we follow the evaluation protocol used in RSL-SQL^31^and introduce two recall-based metrics:

Non-Strict Recall (*NSR*): *NSR* measures the degree of overlap between the schema elements retrieved by the framework and the schema elements actually involved in the gold SQL. It is defined as the total number of overlapping elements between the retrieved and ground-truth schema across all queries, divided by the total number of ground-truth schema elements. The formula is:

1 $$\begin{aligned} NSR = \frac{\sum _{i=1}^{n} \left| S_{gt,i} \cap \overline{S}_i \right| }{\sum _{i=1}^{n} \left| S_{gt,i} \right| } \end{aligned}$$

where *n* is the number of questions, $S_{gt,i}$ denotes the set of schema elements involved in the gold SQL for the *i*-th question, and $S_i$ denotes the set of schema elements retrieved by the framework for that question.

Strict Recall Rate (*SRR*): *SRR* represents the proportion of questions for which the framework successfully recalls all required columns involved in the gold SQL. If the framework correctly recalls all the schema elements for a query, it is counted as 1; otherwise, it is counted as 0. The formula is:

2 $$\begin{aligned} SRR = \frac{\sum _{i=1}^{n} \mathbb {I}(\overline{S}_i \supseteq S_{gt,i})}{n} \end{aligned}$$

$\mathbb {I}(\cdot )$ is the indicator function that returns 1 if the condition holds, and 0 otherwise.

### Baseline methods

In our experiments, we compared Dynamic-SQL with several state-of-the-art approaches in the NL2SQL field on the BIRD dataset. These baseline methods adopt various strategies to enhance performance and represent the cutting edge of NL2SQL research.

RSL-SQL^31^ consists of four main components: bidirectional schema linking, contextual information enhancement, binary selection strategy, and multi-round self-correction mechanism. Its pruning strategy, which combines forward and backward directions, significantly improves schema linking recall.

DIN-SQL^21^ includes schema linking, query classification and decomposition, SQL generation, and self-revision. This method demonstrates that when tasks are properly decomposed, large language models (LLMs) can effectively solve each subproblem.

MAC-SQL^19^ constructs a multi-agent collaborative framework comprising a decomposer, selector, and optimizer. By leveraging specialized cooperation, it improves both the accuracy and efficiency of SQL generation.

CHESS^30^ is an end-to-end system designed for complex databases. It integrates entity context retrieval, schema selection, and SQL generation into a joint optimization pipeline.

These baseline methods reflect the frontier of NL2SQL research from different perspectives, including task decomposition, agent collaboration, end-to-end modeling, and multi-candidate optimization.

### Experimental results

**Table 1:** Comparison of the recall rates NSR and SRR of pattern links of different methods on the BIRD dataset, where Avg T and Avg C are the average number of recalled tables and columns, respectively.

**Methods**	**Model**	**NSR**	**SRR**	**Avg T**	**Avg C**
Full Schema	-	100	100	7.44	76.28
Gold SQL	-	100	100	1.94	4.74
CHESS^30^	-	94.00	89.70	1.92	4.47
RSL-SQL^31^	DeepSeek	97.29	93.28	5.36	14.85
RSL-SQL^31^	GPT-4	97.69	94.32	5.40	13.02
RSL-SQL^31^	qwen2.5-coder-32b-instruct	95.78	85.92	5.35	10.78
Dynamic-SQL	qwen2.5-coder-32b-instruct	95.61	90.66	5.24	16.10
Hybrid Retrieval	-	84.41	62.20	5.40	13.84
Initial SQL Generation	qwen2.5-coder-32b-instruct	88.93	72.63	1.94	4.77
Dynamic-SQL	qwen2.5-coder-7b-instruct	94.18	83.25	5.24	16.01
Hybrid Search	-	84.41	62.20	5.40	13.84
Initial SQL Generation	qwen2.5-coder-7b-instruct	80.12	57.69	1.89	4.51

We conducted comprehensive experiments on the BIRD dataset using two open-source models: Qwen2.5-Coder-32B-Instruct and Qwen2.5-Coder-7B-Instruct. By comparing with various baseline methods described in Section 4.3, we systematically evaluated the performance of the Dynamic-SQL framework from two main perspectives: schema linking performance and SQL execution accuracy. Table 1 focuses on schema linking and compares recall rates across methods. Table 2 presents the overall performance on the Text-to-SQL task, detailing execution accuracy across varying levels of query complexity in the BIRD dataset.

#### Schema linking performance analysis

In Text-to-SQL tasks, the quality of schema linking directly affects both the accuracy of the final SQL query and the prompt length. To thoroughly assess the schema linking performance of Dynamic-SQL, we conducted experiments on the BIRD dataset and compared NSR and SRR against existing methods.

As shown in Table 1:CHESS performs schema linking through multiple rounds of LLM-based filtering on top of retrieval, achieving an SRR of **89.7%**.RSL-SQL employs a bidirectional schema linking strategy with two LLM invocations and achieves impressive performance: **94.32%** SRR with GPT-4, **93.28%** with DeepSeek-Coder, and **85.92%** with Qwen2.5-Coder-32B-Instruct.

In contrast, Dynamic-SQL performs schema linking with only a single LLM invocation. By combining hybrid vector retrieval and LLM generation guidance, it addresses the common issue of “high redundancy but insufficient recall” in existing methods. It achieves **90.66%** SRR on Qwen2.5-Coder-32B-Instruct, while reducing the average prompt length by **50.83%**, striking a better balance between accuracy and efficiency–surpassing both CHESS and RSL-SQL under the same model settings.

The results show that the hybrid retrieval strategy used by Dynamic-SQL ensures broad initial coverage. Although some redundancy and omissions remain, these are effectively addressed by the guided SQL generation phase, which extracts fields relevant to the query. The union of retrieval and generation results forms a concise schema subset with essential information and minimal redundancy.

In summary, the experiments demonstrate the high complementarity of the retrieval-generation cooperative strategy. Hybrid retrieval offers broad candidate coverage, while LLM-guided generation performs semantic-level refinement and completion. This strategy not only boosts recall but also significantly reduces input costs, making it key to the efficiency and robustness of Dynamic-SQL schema linking.

#### Comparison between Best-of-N and Multi-Path Chain-of-Thought Fusion

In this experiment, we compare the performance of Best-of-N and Multi-Path Chain-of-Thought Fusion (MPCF) approaches. For each query, multiple SQL candidates are generated using both methods. The Best-of-N approach selects the highest-scoring candidate from the set, while MPCF integrates multiple reasoning paths by combining execution feedback and structural comparisons to generate the final SQL query. We use execution accuracy as the sole evaluation metric.

**Table 2:** Execution accuracy of Best-of-N and Multi-Path Chain-of-Thought Fusion on the BIRD dataset at different levels of difficulty.

**Models**	**Methods**	**Execution Accuracy**			**Total**
		**Simple**	**Moderate**	**Challenging**	
qwen2.5-coder-32b-instruct	$Best-of-N$	68.97	53.21	45.51	60.17
	*MPCF*	69.73	53.88	48.97	62.97
qwen2.5-coder-7b-instruct	$Best-of-N$	52.76	36.68	35.86	45.47
	*MPCF*	55.46	37.93	34.48	48.17

As shown in Table 2, Multi-Path Chain-of-Thought Fusion (MPCF) consistently outperforms the Best-of-N method in terms of execution accuracy across all difficulty levels. The qwen2.5-coder-32b-instruct model shows an improvement in execution accuracy from 60.17% (Best-of-N) to 62.97% (MPCF), with the largest gain observed in the challenging queries (3.46% improvement). Similarly, the qwen2.5-coder-7b-instruct model achieves an increase from 45.47% (Best-of-N) to 48.17% (MPCF), with a notable improvement in the simple queries (2.7% improvement).

The results clearly indicate that MPCF is more effective at handling complex queries and producing more semantically consistent SQL queries. The fusion of multiple reasoning paths helps overcome the limitations of the Best-of-N approach, which only selects a single candidate and may miss out on valuable insights from other generated SQLs.

#### Execution Accuracy (EX) on the BIRD dataset

**Table 3:** Comparison of execution accuracy EX of different methods on BIRD dataset.

**Methods**	**Models**	**Execution Accuracy**			**Total**
		**Simple**	**Moderate**	**Challenging**	
MAC-SQL^19^	GPT-3.5	-	-	-	50.56
MAC-SQL^19^	GPT-4	65.73	52.69	40.28	59.39
DIN-SQL^21^	GPT-4	-	-	-	50.72
CHESS^30^	Openllms	-	-	-	61.50
CHESS^30^	Proprietary	-	-	-	65.00
RSL-SQL^31^	DeepSeek	69.73	54.09	54.48	63.56
RSL-SQL^31^	GPT-4o	74.38	57.11	53.79	67.21
RSL-SQL^31^	qwen2.5-coder-32b-instruct	68.97	52.16	48.97	61.99
Dynamic-SQL	qwen2.5-coder-32b-instruct	69.95	54.31	48.97	63.23
Dynamic-SQL	qwen2.5-coder-7b-instruct	57.41	40.95	38.62	50.65

To thoroughly evaluate the overall performance of the Dynamic-SQL framework, we compared it against four baseline methods using the BIRD dataset and two open-source models: Qwen2.5-Coder-32B-Instruct and Qwen2.5-Coder-7B-Instruct. Table 3 summarizes the execution accuracy (EX) of SQL queries generated by Dynamic-SQL and other methods across queries of different difficulty levels.

The results show that Dynamic-SQL consistently delivers strong performance across settings: On Qwen2.5-Coder-32B-Instruct, Dynamic-SQL achieves **63.23%** EX, outperforming many GPT-4-based methods. Compared to RSL-SQL **61.99%** under the same model, Dynamic-SQL improves execution accuracy by **1.24%**, validating the effectiveness of its schema linking, example enhancement, and multi-path reasoning mechanisms. Even on the lightweight Qwen2.5-Coder-7B-Instruct, Dynamic-SQL achieves a respectable **50.65%** EX, demonstrating strong generalization and model compatibility. This shows its potential for scalable deployment in resource-constrained scenarios such as edge computing.

The Dynamic-SQL framework not only outperforms multiple GPT-4-based methods while relying solely on open-source models, but also strikes an excellent balance between model invocation cost, execution performance, and deployment adaptability through structural optimization and guided generation strategies. These results highlight that even small-parameter open-source models, when combined with an optimized framework like Dynamic-SQL, can achieve performance comparable to expensive, closed-source models like GPT-4. This breakthrough demonstrates that through thoughtful system design and model architecture, high-performance Text-to-SQL systems can be achieved at a fraction of the cost.

This finding has significant implications for real-world applications, particularly in environments with resource constraints. By utilizing open-source models and optimizing the process, we can achieve both efficient performance and low deployment costs, making NL2SQL technology more accessible and scalable for a broader range of applications.

### Ablation study

**Table 4:** Execution accuracy of Dynamic-SQL framework at different levels of difficulty on BIRD dataset and incremental analysis of each module. Basic Prompt: A minimal instruction that asks the LLM to generate SQL from the NL question and schema without schema-linking heuristics, examples, reasoning paths, or self-correction.

**Models**	**Step**	**Execution Accuracy**			**Total**
qwen2.5-coder-32b-instruct	Basic Prompt	61.95	50.22	42.07	56.52
	*SL*	66.70	50.86	45.51	${59.97}_{\uparrow {\textbf {3.45}}}$
	*EASG*	68.65	53.88	47.59	${62.19}_{\uparrow {\textbf {2.22}}}$
	*MPFR*	69.73	53.88	48.97	${62.97}_{\uparrow {\textbf {0.78}}}$
	*MRS*	69.95	54.31	48.97	${63.23}_{\uparrow {\textbf {0.26}}}$
qwen2.5-coder-7b-instruct	Basic Prompt	43.97	25.97	24.83	36.71
	*SL*	50.38	34.48	25.52	${43.22}_{\uparrow {\textbf {6.51}}}$
	*EASG*	48.86	36.21	35.86	${43.81}_{\uparrow {\textbf {0.59}}}$
	*MPFR*	55.46	37.93	34.48	${48.17}_{\uparrow {\textbf {4.36}}}$
	*MRS*	57.41	40.95	38.62	${50.65}_{\uparrow {\textbf {2.48}}}$

To assess the effectiveness and necessity of each core module in Dynamic-SQL, we conducted ablation experiments using the open-source models *qwen2.5-coder-32b-instruct* and *qwen2.5-coder-7b-instruct*. Table 4 reports execution accuracy (EX) across varying difficulty levels, illustrating how each component incrementally improves SQL generation performance.

**Base Prompt:** The baseline prompt includes the full database schema, the user query, relevant domain knowledge, and explicit instructions. Under this setting, the EX scores for the 32B and 7B models are **56.52%** and **36.71%**, respectively. **Schema Linking(***SL***):** We introduce the hybrid retrieval strategy to select only the complete schema of tables most relevant to the query, replacing the verbose full schema. This compression and noise filtering improves focus. After adding schema linking, EX rises by **4.78%** for the 32B model and **6.51%** for the 7B model, demonstrating that narrowing the input helps the LLM concentrate on core structural information.

**Example-Augmented SQL Generation(***EASG***):** This stage injects three key elements: (1) the schema subset from schema linking to ensure structural precision, (2) real-value exemplars retrieved via sparse retrieval to enhance understanding of data semantics and distributions, and (3) few-shot question–answer pairs retrieved based on the query and initial SQL. This module boosts EX by **2.22%** (32B) and **0.59%** (7B). Notably, on the Challenging tier the 7B model gains **10.34%**, highlighting the critical role of exemplar augmentation for smaller models and hard cases.

**Multi-Path Chain-of-Thought Fusion and Reasoning(***MPFR***):** We replace the standard best-of-$k$ decoding with our fusion mechanism. By comparing structure and execution feedback across multiple SQL candidates and fusing their reasoning chains, we form a single, semantically coherent query. This improves EX by **4.36%** for the 7B model–especially on Moderate tasks–and by **0.78%** overall for the 32B model (with a **2.22%** gain on Challenging tasks). These gains show that multi-path fusion enhances both semantic consistency and robustness in complex reasoning. **Multi-Round Self-Correction(***MRS***):** Finally, we introduce our iterative self-correction module. When execution fails or returns empty results, the framework reruns fusion over the failed candidates using execution feedback, reconstructing a new SQL. This further raises EX and, in difficult cases, sharply reduces erroneous queries, enhancing the final output’s robustness and fault tolerance.

In summary, each module–schema linking, exemplar augmentation, multi-path fusion, and multi-round self correction contributes substantively to Dynamic-SQL’ accuracy and reliability across all difficulty levels, offering strong technical support for future NL2SQL research.

## Discussion

In this paper, we propose a schema linking method that integrates hybrid vector retrieval and structure-guided generation to enhance the structural perception and reasoning capabilities of LLMs in the Text-to-SQL task. The method consists of three stages: First, a hybrid retrieval space is constructed by combining dense vectors for semantic retrieval and sparse vectors for precise matching, from which the most relevant tables and columns are retrieved for the natural language query. Next, based on the complete schema information of the retrieved tables, the LLM is guided to generate an initial SQL query, which is then reverse-parsed to extract the fields used, further completing the schema information and compensating for any missing fields from the vector retrieval. Finally, the schema information extracted in both stages is fused, and an externally key-referenced semantic and structurally complete schema sub-graph is constructed, which serves as the prompt information for final SQL generation. This strategy effectively compresses the prompt length in complex database environments, reducing redundant structural information and significantly lowering LLM invocation and computational costs. Meanwhile, the structural priors and generation validation feedback loop improve schema linking accuracy, coverage, and robustness, thereby enhancing the quality and interpretability of the final SQL generation.

To address input redundancy, semantic ambiguity, and resource constraints in real-world NL2SQL scenarios, we propose Dynamic-SQL, an NL2SQL framework based on open-source LLMs. Dynamic-SQL integrates a hybrid vector retrieval and LLM-guided schema linking strategy, real-value and few-shot exemplar augmentation, multi-path chain-of-thought fusion reasoning, and execution-driven multi-round self-correction, collectively enhancing the structural integrity and semantic consistency of generated SQL.

Experiments on the BIRD benchmark demonstrate that Dynamic-SQL, powered by *qwen2.5-coder-32b-instruct*, achieves **63.23%** execution accuracy and raises strict schema-linking recall from **72.63%** to **90.66%**, outperforming several GPT-4–based proprietary methods. These results showcase its robustness, scalability, and cost-effectiveness. Ablation studies further quantify the incremental gains of each module across varying difficulty levels, confirming their synergistic contributions to SQL quality.

A key takeaway from these results is that even small-parameter open-source models can achieve performance on par with larger, more expensive models like GPT-4 when combined with an optimized framework like Dynamic-SQL. This finding underscores the potential for achieving high-performance NL2SQL systems that are both cost-effective and scalable, making them suitable for resource-constrained environments such as edge computing or real-time applications. By carefully optimizing the model architecture and system workflow, we demonstrate that high-quality SQL generation can be achieved without relying on costly proprietary models.

In summary, Dynamic-SQL overcomes key limitations of open-source models in complex schema understanding and semantic parsing, and offers a practical path to deploy high-performance NL2SQL systems under resource constraints. Future work will explore more efficient vector retrieval mechanisms to boost schema recall, optimize the multi-round feedback loop for production-grade latency, and extend the framework to cross-lingual, multimodal database interactions and complex business-logic scenarios, paving the way for large-scale enterprise-level deployments.

## Methods

### Prompt engineering

When applying LLMs to the NL2SQL task, prompt design is critical. High-quality prompts not only significantly impact SQL generation accuracy but also determine the transferability and generalization of the Dynamic-SQL framework across different database schemas. In this work, we focus on the following key input elements:

1. **User Query(***q***):** The natural-language request expressed by the user, serving as the primary semantic input for the NL2SQL task.
2. **Database Schema Information(***s***):** Following the M-Schema representation in Xiyan-SQL^35^, we provide a concise semi-structured description of each table’s name, column names, data types, and foreign-key relationships. This ensures that the generated SQL queries reference the correct tables and columns, and aids the framework’s generalization to diverse schemas.
3. **Column Descriptions and Enumerated Values(***d***):** For each column, we supply a brief semantic description and, where applicable, a list of its possible values. This helps the LLM better understand column semantics and improves the accuracy of column matching.
4. **Value Samples(***val***):** Using a sparse-vector retrieval strategy, we extract the top real-world values most relevant to the user query from a vector store, and present them as examples to enhance the LLMs’ awareness of column semantics and data distributions. For columns without sparse representations, we randomly sample five records from the corresponding table, truncating overly long text values as needed.
5. **Additional Context(***k***):** Any other task-relevant prior knowledge–such as business rules, domain-specific terminology definitions, or historical queries–is included to improve the LLMs’ scene understanding and ensure consistency between generated SQL and the application context.

### Schema linking

**Fig. 2:**
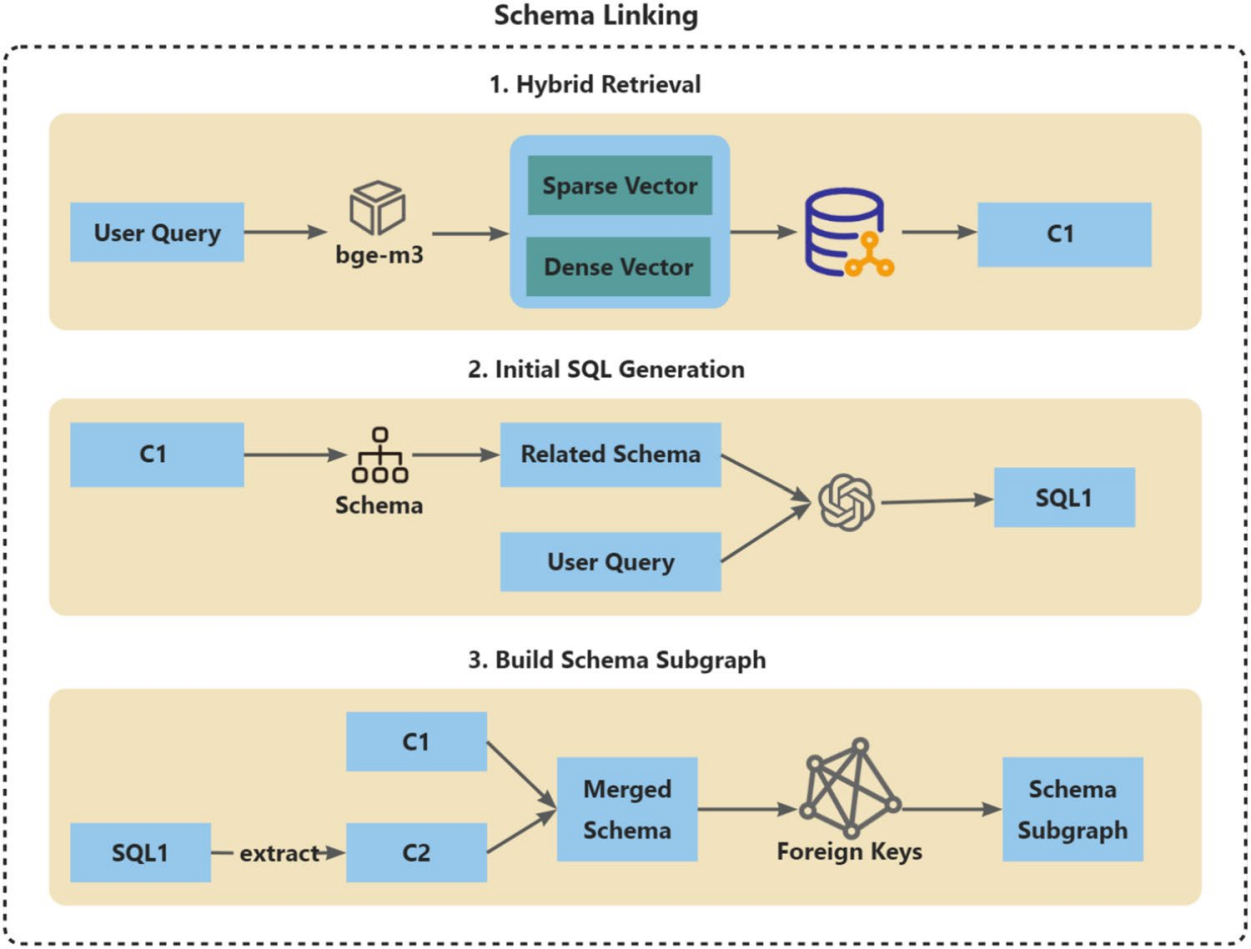
Overall Schema Linking Architecture.

Schema linking aims to establish high-quality mappings between the semantic elements in a natural-language query and the database’s tables and columns, thus providing a structured semantic foundation for SQL generation. Unlike traditional pipeline methods that “match schema first, then generate SQL,” Dynamic-SQL introduces a generation-guided structure recognition strategy (Fig. 2). First, a dense–sparse hybrid vector retrieval mechanism recalls candidate tables and columns most relevant to the query from a vector store. Next, using the full schema of these candidates, an LLM is prompted to generate an initial SQL query. Finally, schema information is reverse-parsed from the generated SQL and fused with the initial retrieval results; foreign-key metadata are used to complete join paths and construct a minimal, closed sub-schema graph. This approach mitigates semantic uncertainty and structural noise caused by overly long prompts in complex databases, substantially improving structure identification accuracy and generalization.

#### Vector space construction and hybrid retrieval

To achieve multi-level understanding of query semantics, we build a multi-granularity vector representation that fuses schema metadata with real data values. Specifically:

**Dense Vector Space:** For the three types of structured metadata in the database–field name $f^1$, field description $f^2$, and value description $f^3$ –a multi-channel semantic encoding approach is used. Leveraging the BGE-m3 model, deep semantic modeling is performed to generate a dense vector $v_d$:

3 $$\begin{aligned} v^i_d = BGE - m3_{\text {dense}} (f_i^1, f_i^2, f_i^3). \end{aligned}$$

This representation has strong capabilities in synonym recognition and cross-domain transfer, providing foundational support for retrieving semantically similar fields.

**Sparse Vector Space:** For columns $f^{value}$ in the database that contain rich yet sparsely distributed information–such as enumerated field values or short text values–a term-driven sparse vector representation is constructed, emphasizing keyword matching and frequency-awareness. The sparse vector $v_s$ is built based on the term-level semantic scores generated by the BGE-m3 model:

4 $$\begin{aligned} v^i_s = BGE - m3_{\text {sparse}} (f_i^{value}). \end{aligned}$$

The sparse space is better suited for capturing local statistical features such as frequency patterns and value distributions, thereby compensating for the limitations of dense vectors in precise matching.

**Hybrid Retrieval and Fusion Ranking Mechanism:**To leverage the complementary strengths of the two vector spaces, a multi-channel hybrid retrieval mechanism is adopted. For the dense vector space (3 channels), semantic retrieval is performed based on cosine similarity; for the sparse space (1 channel), a sparse recall mechanism driven by inner product is used. Each returns a candidate set of fields independently. Subsequently, the *RRFRanker* module is used for fusion ranking, where multi-channel scores are normalized and aggregated to produce the final ranking result:

5 $$\begin{aligned} c_1 = Top-k(COSINE(v_d^q,v_d)+IP(v_s^q,v_s)) \end{aligned}$$

This approach yields a ranked list of candidate fields that combines both semantic

#### Initial SQL generation

After obtaining the candidate field set $c_1$, the complete schema information of the corresponding tables (including table names, fields, data types, and foreign key constraints) is extracted to construct a semi-structured candidate schema set $s_1$. At the same time, field descriptions and enumerated value sets $d_1$, along with other contextual information $k_1$, are integrated as prompt context and jointly fed into the large language model $f_{LLM}$ to generate the initial SQL query $sql_1$:

6 $$\begin{aligned} sql_1 = f_{LLM}(q,s_1,d_1,k_1) \end{aligned}$$

The goal of this stage is to leverage the global reasoning capability of the LLM to generate an initial SQL query that is structurally complete, logically coherent, and semantically consistent based on the full schema information of the candidate tables. The purpose is to fully activate the LLMs’ ability to understand and compose database structures, rather than directly producing the final executable SQL query.

#### SQL-driven subschema backward construction

After obtaining $sql_1$, structural parsing is performed to extract all referenced table names and column names, forming a generation-driven candidate field set $c_2$. To further enhance the accuracy and completeness of schema linking, $c_2$ is merged with the initially retrieved candidate field set $c_1$:

7 $$\begin{aligned} c_{merge}=c_1 \cup c_2 \end{aligned}$$

Then, based on the foreign key dependencies *FK* in the database, the table join paths are completed to ensure structural connectivity among the fields. This process produces a minimal subschema graph $s_{min}$ that is structurally closed and semantically coherent. This subschema serves as the final output of the schema linking stage and acts as the foundational structural input for the subsequent stages of SQL generation, chain-of-thought reasoning, and self-correction, satisfying three key constraints: semantic relevance, structural closure, and execution feasibility:

8 $$\begin{aligned} s_{min}=closure(FK,c_{merge}) \end{aligned}$$

This section establishes a high-quality mapping from natural language to structural context through several stages, including hybrid vector retrieval, generation-guided structural recognition, and SQL-based reverse subschema construction. This strategy not only improves the robustness of schema linking but also significantly enhances the structural awareness of the LLM, laying a solid foundation for downstream SQL generation and reasoning.

### Example-augmented SQL generation

**Fig. 3:**
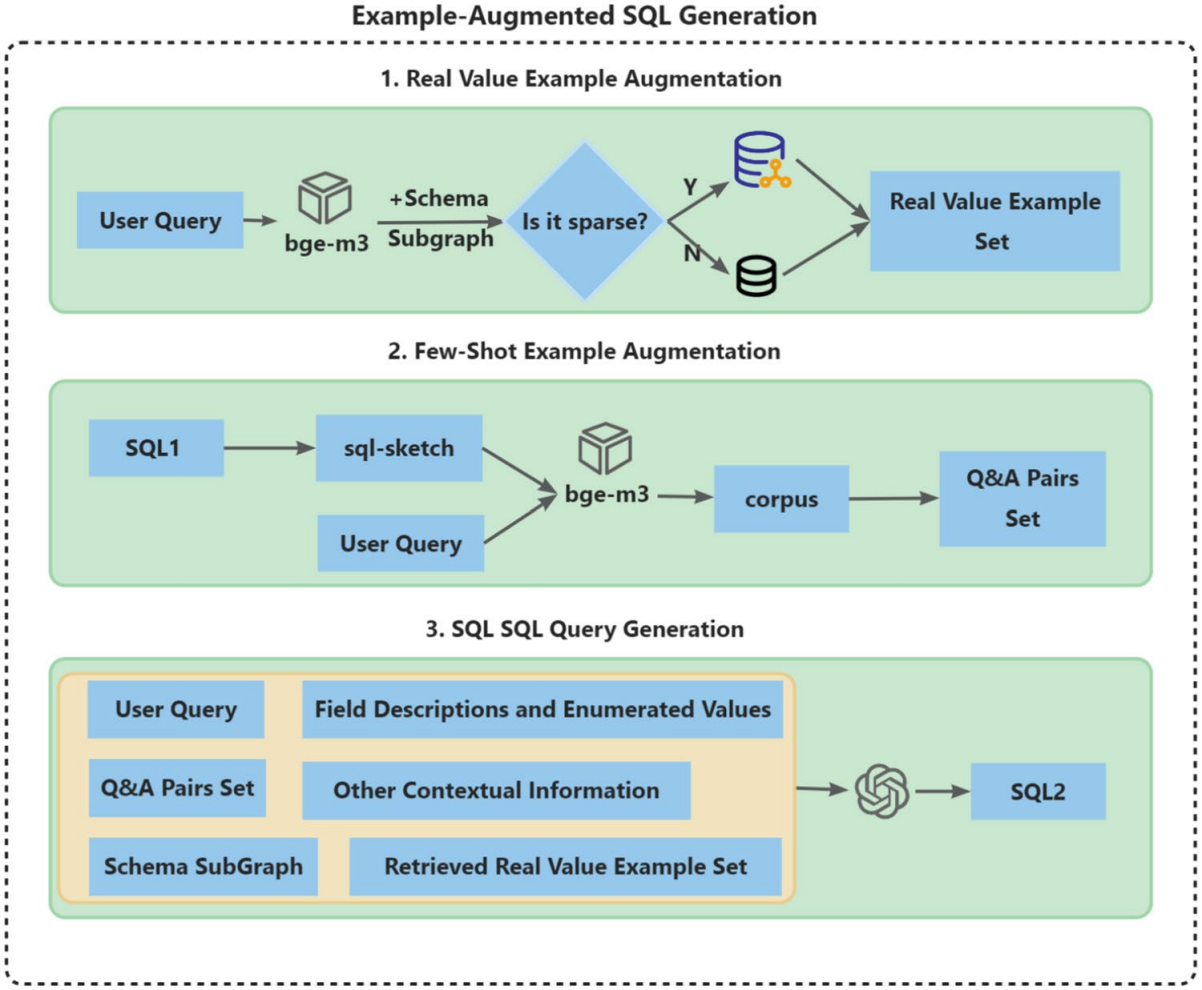
Example-Augmented SQL Generation Architecture Diagram.

After completing schema linking, the Dynamic-SQL framework enters the stage of generating SQL queries based on the structural context. Existing approaches mostly rely on table schemas and natural language prompts for SQL generation but often overlook the crucial role of real field values in the semantic reasoning process. This can lead to misjudgments or omissions by the LLM in condition construction, entity recognition, and constraint expression. To address this issue, this section introduces two example enhancement mechanisms, as shown in Fig. 3: Incorporating real-value examples that are highly semantically relevant to the current query^36^; Incorporating high-quality QA pairs that are highly similar to the current query in both semantics and structure as few-shot examples^37^.These mechanisms effectively alleviate issues such as ambiguity, constraint misjudgment, and difficulty in entity resolution during the reasoning process, thereby improving the semantic alignment and structural validity of the generated SQL.

#### Real-value example enhancement mechanism

Based on the subschema $s_min$, this mechanism utilizes the sparse vector space to retrieve real-value examples most relevant to the query semantics, thereby enriching the structural context and enhancing the LLMs’ understanding of field semantics, entity types, value preferences, and constraint boundaries. Unlike global retrieval, the examples are searched only within the identified field set $c_merge$. For each candidate field $f_i$, the user query *q* is first encoded as a sparse vector $v_d^s$ using the BGE-m3 model. Then, inner product calculations are performed between $v_d^s$ and the vectors in the sparse space to compute semantic relevance between the query and the field values:

9 $$\begin{aligned} val_j=top-k(IP(v_s^q,v_s)) \end{aligned}$$

Field values with higher scores are more likely to be semantically relevant to the query and are prioritized for inclusion in the prompt context.

The example construction strategy varies by field type: For enumerated fields, all possible values from the database are extracted to help the LLM understand the complete semantic range of type- or state-related fields.For short text fields, 1 to 5 real value samples that are most semantically relevant to the query are selected to enhance the LLMs’ understanding of entity references within the query.For numeric fields, long texts, or others not suitable for sparse modeling, 5 sample values are randomly drawn from the database to help the LLM grasp the value range and structural characteristics of the field, with overly long values appropriately truncated.

By introducing real-value examples highly relevant to the question, this mechanism provides semantic anchors for the LLM. It not only improves the alignment between fields and query intent but also supplies valuable context for subsequent SQL generation.

#### Few-shot example enhancement mechanism

To further improve the semantic alignment and structural generalization of SQL queries, Dynamic-SQL introduces a structure-aligned few-shot example enhancement mechanism, which selects high-quality QA pairs that are highly similar to the current query in both semantics and structure. Based on a unified representation alignment strategy, high-quality QA pairs are retrieved from a preprocessed example corpus and embedded into the prompt as few-shot examples. This helps the LLM better understand the structural patterns and semantic boundaries of the current query.

To achieve both structural and semantic alignment, a unified vector representation and multi-stage matching process is designed. First, the initial SQL query $sql_1$, generated based on the subschema, is parsed to extract its structural features–such as involved tables, JOIN structure, number of WHERE conditions, presence of aggregation or nesting–and a standardized structure sketch $sql-sketch$ is constructed. This structure sketch is then concatenated with the original natural language query to form a structure-semantic description pair, which is encoded into a unified vector representation using the BGE-m3 model:

10 $$\begin{aligned} v_qs = BGE-m3(q, sql-sketch) \end{aligned}$$

Similarly, during offline preprocessing, all QA pairs in the corpus are processed in the same way: structural sketch extraction and unified vector encoding, forming the example vector corpus *corpus*. Finally, inner product similarity is computed to retrieve top-k examples that are well-aligned in both structure and semantics:

11 $$\begin{aligned} qs = top-k(IP(v_{qs},corpus)) \end{aligned}$$

These examples are embedded into the prompt as few-shot demonstrations to assist the LLM in generating SQL. Compared with traditional static examples or purely semantic matching strategies, Dynamic-SQL significantly enhances the LLMs’ generalization and structural transfer capabilities in complex queries through its “structure alignment + semantic analogy” approach.

#### SQL query generation

To further improve the execution accuracy of SQL statements, Dynamic-SQL designs chain-of-thought (CoT) prompting templates to guide the LLM in progressively reasoning through the logical relationships among fields, value ranges, and condition combinations–thereby reinforcing logical consistency and deep semantic reasoning.

Finally, a structured input is constructed by combining the few-shot examples *qs*, real-value example set *val*, user query *q*, subschema $s_min$, field descriptions and enumeration sets $d_1$, and additional context features $k_1$. This composite prompt is fed into the large language model $f_LLM$, which generates a structurally sound, semantically precise, and constraint-complete SQL query $sql_2$:

12 $$\begin{aligned} sql_2=f_{LLM}(q,s_{min},d_1,val,k_1,qs) \end{aligned}$$

By incorporating subschema structure, real-value examples, and structurally aligned QA pairs, the LLM is able to produce high-quality SQL queries that are semantically accurate, structurally complete, and directly executable–providing strong candidates for the subsequent reasoning and self-correction stages.

### Multi-path chain-of-thought fusion and reasoning

After generating the initial SQL query ($sql_1$) and the example-enhanced SQL query ($sql_2$), the Dynamic-SQL framework introduces a multi-path chain-of-thought fusion mechanism to achieve deeper structural alignment and semantic integration. Unlike traditional Best-of-N strategies, which merely select the highest-scoring candidate from multiple independent SQLs and often fail to leverage the structural and semantic complementarity between them^38^, our approach adopts a fusion-based generation paradigm. This approach guides the large language model (LLM) to perform structural comparison, semantic integration, and reasoning across multiple SQL candidates, ultimately generating a new SQL query ($sql_3$) that exhibits higher semantic alignment and stronger execution robustness.

Specifically, the LLM compares structural differences among candidate SQLs (such as field selection, join paths, and filtering conditions), aligns them, and incorporates execution feedback from each candidate based on real database interactions. This comparison helps the model merge and reconstruct a new SQL query ($sql_3$) that retains effective structures while minimizing semantic deviations. Instead of simply selecting the best candidate, the model performs multi-path fusion reasoning and semantic reconstruction, fully extracting and refining the effective structures and conditions embedded in each SQL path.

**Fig. 4:**
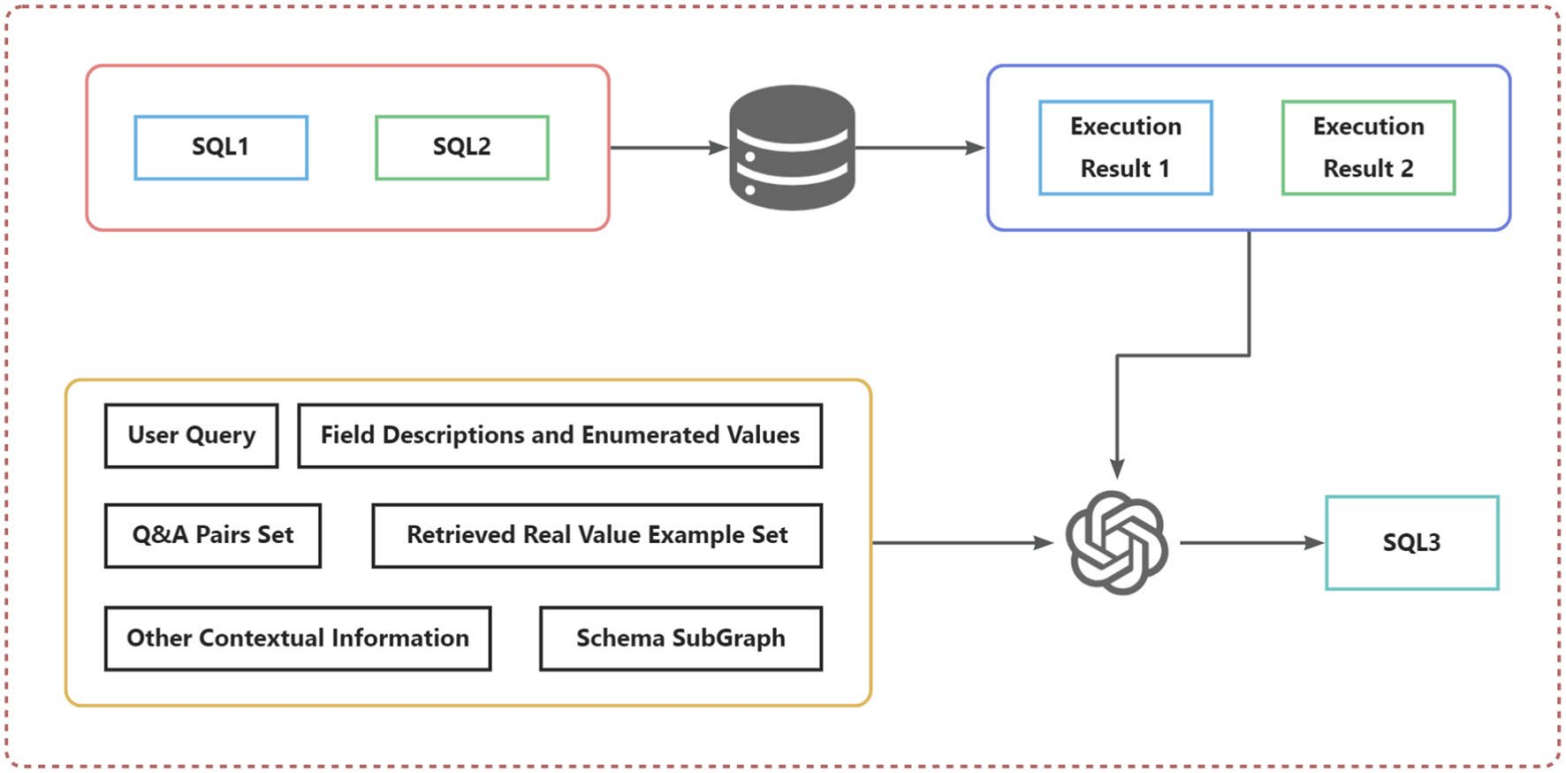
Multi-Path Chain-of-Thought Fusion and Reasoning Flowchart.

To facilitate this process, Dynamic-SQL constructs specialized **multi-path chain-of-thought prompts** (Fig. 4). These prompts combine the user query *q*, the relevant sub-schema $s_{min}$, field descriptions and enumeration values $d_{min}$, real-value examples *val*, and contextual features $k_1$. Along with the initial SQL queries $sql_1$ and $sql_2$ generated in previous stages, the execution feedback $r_1$ and $r_2$ corresponding to each SQL query are also included in the input. This complete prompt is passed to the LLM, which performs fusion reasoning and generates a refined SQL query:

13 $$\begin{aligned} sql_3 = f_{LLM}(q, s_{min}, d_{min}, val, k_1, qs, (sql_i, r_i)_{i=1}^2) \end{aligned}$$

The execution feedback $r_i$ can either be the result of a successful query execution (possibly truncated to control prompt length) or an error type and message if the execution fails. The fusion process follows a **chain-of-thought reasoning** path based on structural comparison, feedback analysis, and logical synthesis. This guides the LLM to extract effective substructures from different candidate SQLs and revise potential semantic inconsistencies using execution feedback. The result is a final SQL query that is structurally concise, semantically accurate, and highly executable–providing a stronger initial input for the subsequent self-correction phase.

### Multi-round self-correction

Although the fused SQL query $sql_3$ already exhibits high consistency and executability in terms of structure and semantics, it may still fail during execution or return empty results in a real database environment due to subtle semantic deviations or logical conflicts. To address this, Dynamic-SQL introduces a multi-round self-correction mechanism. This mechanism is driven by execution feedback, forming an iterative, feedback-enhanced SQL refinement process to further improve the query’s executability and robustness.

In this stage, $sql_3$ is first executed. If it succeeds and returns non-empty results, it is directly used as the final output. Otherwise,if the query execution fails or the result is empty-the system captures the execution feedback (e.g., error type, error message, or contextual information about the empty result), denoted as $e^0$, and triggers the self-correction mechanism, entering an iterative optimization loop based on multi-path fusion.

This process reuses the multi-path chain-of-thought prompt structure from the previous stage, dynamically incorporating each round’s historical SQL paths and corresponding execution feedback into the prompt. This guides the LLM to perform semantic corrections and logical reconstruction based on prior reasoning paths. The optimization process is represented as:

14 $$\begin{aligned} sql_4^{(i+1)}=f_{LLM}(q,s_{min},d_1,val,k_1,qs,(sql_j,r_j)_{j=1}^2,(sql_4^t,e^t)_{t=0}^i) \end{aligned}$$

Here, $sql_3$ is considered the initial input and denoted as $sql_4^0$, while $sql_4^i$ refers to the cumulative set of SQLs generated up to iteration *i*. $e^t$ denotes the associated execution feedback (e.g., empty result or specific error type). The self-correction loop terminates when a generated query $sql_4^i$ executes successfully and returns non-empty results, or when the maximum number of iterations *N* is reached. The final query is denoted as $sql_{final}$.

This optimization process integrates path accumulation and semantic evolution mechanisms. In each round, the LLM performs chain-of-thought reasoning to compare differences across SQL queries-such as field selection, join structures, and condition expressions-extracting high-quality substructures. These are refined using execution feedback to perform logical correction and semantic reconstruction, ultimately producing an SQL query that is more concise in structure and more aligned in meaning.

## Data Availability

The BIRD-bench dataset is publicly available at https://bird-bench.github.io/under the CC BY-SA 4.0 license. The raw data were not further processed.

## Code availability

All relevant source code is available at https://github.com/ChIIChI1230/Dynamic-SQL.
